# Psychoeducational Intervention for Perinatal Depression: Study Protocol of a Randomized Controlled Trial

**DOI:** 10.3389/fpsyt.2019.00055

**Published:** 2019-02-13

**Authors:** Luca Steardo, Vito Caivano, Gaia Sampogna, Arcangelo Di Cerbo, Giovanna Fico, Francesca Zinno, Valeria Del Vecchio, Vincenzo Giallonardo, Marco Torella, Mario Luciano, Andrea Fiorillo

**Affiliations:** ^1^Department of Psychiatry, University of Campania Luigi Vanvitelli, Naples, Italy; ^2^Department of Gynecology, University of Campania Luigi Vanvitelli, Naples, Italy

**Keywords:** perinatal depression, women mental health, severe mental disorders, family burden, coping strategies

## Abstract

Perinatal depression (PD) is a severe and disabling condition impacting negatively on children in terms of adverse neonatal outcomes and on the well-being of women and their families. All pregnant women attending the unit of Gynecology and Obstetrics Service of the University of Campania “L. Vanvitelli” will be screened for PD using the Edinburgh Postpartum Depression Scale (EPDS). Women with a score ≥10 at the EPDS will be invited to receive a full psychiatric assessment. The required sample size is of 126 women with PD which will be randomly allocated to either an experimental group, receiving a uni-familiar psychoeducational intervention, or to a control group, receiving the Best Treatment Option (BTO). Patients will be evaluated through several assessment instruments: Hamilton Depression Rating Scale (HAM-D), Hamilton Anxiety Rating Scale (HAM-A), Global Assessment of Functioning (GAF), Clinical Global Impression (CGI), Manchester Short Assessment of Quality of Life (MANSA), Family Assessment Device (FAD), Family Coping Questionnaire (FCQ), and Pattern of Care Schedule (PCS). Patients will be evaluated at baseline, 3, 6, 9, and 12 months post-randomization. The severity of depressive symptoms at the HAM-D scale has been selected as primary outcome. Other outcome measures include improvement in the severity of anxiety symptoms, of global and personal functioning, an improvement in family members' coping strategies and in the level of quality of life. It has been highlighted the importance of developing screening and treating programs for PD, and our study will use rigorous study design to evaluate the efficacy of the adaption of a well-known family psychoeducational model to the treatment of PD. The aims of present trial are to: (1) develop an informative package for pregnant women with PD; (2) promote a screening programme for PD; (3) identify those (socio-demographic and pregnancy-related environmental) factors associated with a higher risk to develop a perinatal or postnatal depression; (4) evaluate the efficacy of a new experimental psychoeducational intervention in reducing the depressive symptoms during pregnancy compared to the BTO.

## Introduction

Depressive disorders represent the major cause of disability worldwide (1, 2). These disorders are prevalent in the perinatal period and about 12% of women are affected (3). However, prevalence rates of perinatal depression (PD) should be cautiously considered since this disorder is frequently underdiagnosed, mainly because patients experience delays and difficulties in help-seeking due to feelings of guilty and fear of stigmatization (4, 5). PD poses a significant burden on affected women, their families and on society at large; moreover, it carries serious long-term consequences on the mental health of the new born (6, 7). In particular, PD can have several detrimental effects including low birth weight, preterm birth, small development for gestational age, early childhood developmental delays, poor maternal fetal attachment, impairments in cognitive functioning, behavioral disturbances, and development of depressive disorders in the childhood or adolescence (8–16).

Different risk factors have been identified for PD, including low socioeconomic status (17–19), being a single mother (17, 20), poor social support (21), general life stress (17), and unplanned pregnancies (22). Indeed, PD is associated with a dysregulation and hyperactivity of the hypothalamic-pituitary-adrenal axis activity (HPA) (23), with an increased exposition to the corticotrophin-releasing hormone (CRH) during intrauterine development.

Several interventions have been developed for reducing the impact of risk factors and for preventing the development of PD (24), including professionally-based home visits, postpartum peer-based telephone support, interpersonal psychotherapy (25), and cognitive behavioral therapy (26). Although several of these interventions have been made available in clinical practice, research is needed in order to confirm their efficacy (27).

Symptoms of perinatal depression often include anxiety, irritability, sleep disturbance, low mood, and excessive concern for the child's care (28), in fact, PD is a multi-faceted and complex condition which can have heterogeneous clinical presentations (15). In particular, a significant number of women with PD experience comorbid anxiety (29, 30), obsessive-compulsive symptoms and post-traumatic stress disorders (31).

One of the main debated issues regarding the optimal prevention and treatment of perinatal depression is related to the opportunity to early detect the disorder through screening procedures (32, 33). In particular, it has been argued that screening programmes can be effective in reducing burden and disability associated with the disorder (34), although there is the risk to create false-positives (35). Screening procedures aim to detect people at high risk to develop a full-blown disorder, and therefore the clinical utility of the screening process is greatly influenced by the ability to accurately identify those patients (33–35).

However, screening programmes are considered acceptable and even desirable by most pregnant women, both depressed and non-depressed (36). Therefore, available guidelines (37, 38) recommend that healthcare professionals (including midwives, obstetricians, health visitors, and general practitioners) ask questions about past or present mental illness, family history of perinatal mental illness and previous psychiatric treatments including inpatient care. Moreover, the American Academy of Pediatrics also recommend that pediatricians screen new mothers for depression during their visits in the 6 months following childbirth (39).

According to a recent review (40), treatment of PD depends on symptom severity and functional impairment (41, 42). Cognitive behavioral therapy (43), interpersonal psychotherapy, and psychoeducation (44, 45) are usually adopted in case of mild to moderate forms of PD; while antidepressants, more often selective serotonin reuptake inhibitors (SSRI), are used for severe cases (46–51).

When available, pregnant women with PD prefer psychotherapy (52) and the effectiveness of psychological and psychosocial treatments for PD has been explored (53).

Several initiatives have been proposed worldwide in order to assess the efficacy and feasibility of psychoeducational interventions for women with PD. A recent meta-analysis by Sockel (26) found only one study (54) evaluating the efficacy of a psychoeducational programme for women with PD in improving depressive symptoms. More recently, other programmes have been proposed also in low-middle income countries (55).

Psychoeducational interventions are effective in reducing affective symptoms and the levels of stress, with low costs for the mental health department (56).

Based on these premises, the Department of Psychiatry of the University of Campania “Luigi Vanvitelli” is carrying out a study to develop and test the efficacy of a psychoeducational family intervention in pregnant women with perinatal depression and their close relatives.

To our knowledge no randomized controlled trial has been carried out so far in Italy on the effectiveness of psychoeducational interventions for women affected by perinatal depression, although psychoeducational intervention has demonstrated its efficacy in a wide range of severe mental disorders, such as bipolar disorder (57), schizophrenia (58), major depression (59), obsessive compulsive disorder (60), and eating disorders (61).

## Aims

The present trial aims to evaluate the efficacy and feasibility of a new psychoeducational family intervention compared to the Best Treatment Option (BTO) in a sample of women affected by perinatal depression and their family members. The Best Treatment Option (BTO) is provided according to the NICE guidelines (62). In particular, mild to moderate forms of depression may be addressed with self-help or psychological counseling. For moderate or severe forms of PD, high-intensity psychological intervention, or pharmacological treatments are recommended.

The secondary aims are to: (1) improve mental health literacy on the topic of perinatal depression, with a specific focus on risk and protective factors as well as on available therapeutic strategies for the management of perinatal depression; (2) identify clinical, socio-cultural, and pregnancy-related predictive factors for the development of perinatal depression; (3) improve coping strategies and family functioning of family members participating to the intervention; (4) evaluate the long-term effects of the intervention.

## Methods

### Design

This is a randomized controlled trial with two parallel arms for evaluating the efficacy and effectiveness of psychoeducational family intervention in improving depressive symptoms in patients with perinatal depression. The new psychoeducational intervention has been developed by the research staff, based on the Falloon model of psychoeducation for patients with schizophrenia and their families (63).

## Patients' Recruitment Procedure

**Figure 1:**
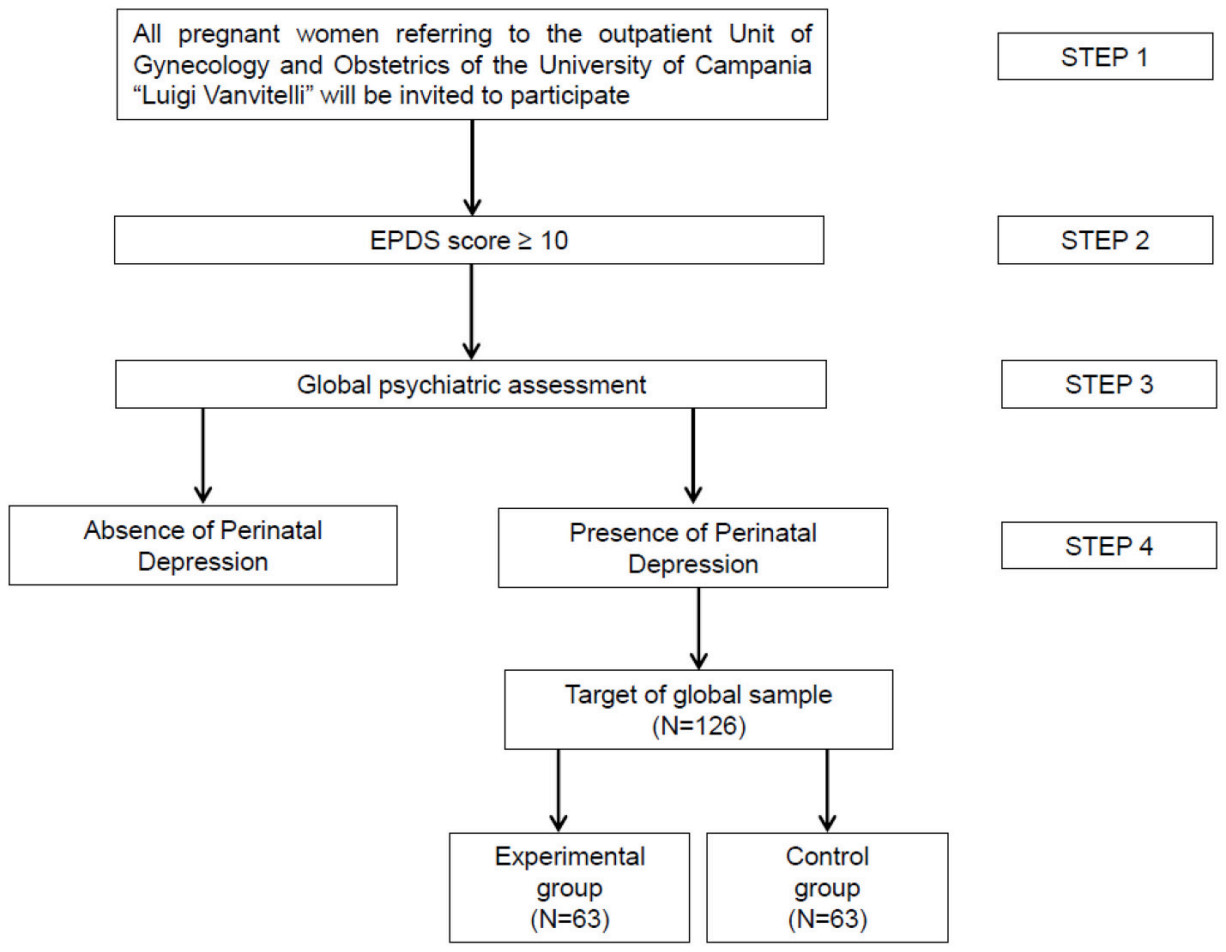
Multi-step recruitment procedure.

Recruitment for the RCT will be conducted adopting a multistep strategy, as reported in Figure 1:

- Step 1. All pregnant women attending the outpatient unit of Gynecology and Obstetrics of the University of Campania “Luigi Vanvitelli” will be invited to participate in the study through the use of informative leaflets on perinatal depression (i.e., characteristics of the disorder, risks, long-term consequences, available treatments). Those interested in having more information will be referred to a research team member presenting a brief overview of study procedures and time commitment in order to obtain their informed consent. Once informed consent is obtained, patients will be officially enrolled in the study and will undergo the screening procedure, compiling the Edinburgh Postnatal Depression Scale (EPDS) (64).
- Step 2. Women with a score ≥10 at the EPDS will be invited to receive a full psychiatric assessment.
- Step 3. Patients will receive a full mental state examination by an expert clinician working at the Department of Psychiatry of the University of Campania “Luigi Vanvitelli,” in order to evaluate the presence of any mental disorder.
- Step 4. Once a diagnosis of perinatal depression is made, patient's code will be given to the statistician for randomization in one of the two groups. Patients will be allocated through a randomized procedure (with a 1:1 ratio) to the experimental or to the control group.

## Inclusion Criteria

Starting from January 2019, patients attending the outpatient unit of Gynecology and Obstetrics of the University of Campania “Luigi Vanvitelli” will be invited to participate to the study. The eligibility criteria will be: (1) gestation age > 3 months or within 3 days of childbirth; (2) age ≥ 18 years; (3) absence of any disabling physical condition; (4) living with a relative for at least 9 months in the last year and continuously in the last 3 months.

## Exclusion Criteria

Exclusion criteria will be: (1) intellectual disability; (2) diagnosis of schizophrenia, schizoaffective disorder, delusional disorder, or other not specified psychosis-spectrum disorder; (3) having experienced depressive symptoms before pregnancy.

## Intervention

### Theoretical Background of the Experimental Intervention

Experimental intervention includes techniques derived from classic psychoeducation (63), motivational intervention (65, 66) and cognitive behavioral therapy (67). The intervention has been developed following the guidelines on the management of perinatal depression released by NICE (62), the American College of Obstetricians and Gynecologists (68) and the United States' Preventive Service Task Force (69). The adopted methodology included the following phases: (1) analysis of the scientific literature; (2) evaluation of available handbooks and manuals of other psychosocial interventions targeting perinatal depression; (3) focus groups with expert researchers, clinicians, users and carers, in order to identify the most relevant information to be included in the intervention; (4) development of an *ad-hoc* manual with a detailed description of each session of the intervention, booklets, and other written materials are provided to patients and relatives, whenever relevant; (5) evaluation of the feasibility of the “pilot” intervention in a small-size study.

### Features of the Experimental Intervention

The intervention consists in a uni-familiar psychoeducational intervention, scheduled every 7–10 days. It includes six modules on:

1. Individual and family assessment. This module is focused on the assessment of patients' personal and social functioning, and on the identification of personal goals. Moreover, the communication skills adopted by family members are evaluated as well as the characteristics of family functioning.
2. Information on the clinical characteristics of the disorder and its treatment. In this module, information on the main clinical and epidemiological features of PD, including incidence, prevalence, long-term outcome, risk for the children, and negative impact on mother's mental health are presented. Moreover, available pharmacological and non-pharmacological treatment options are discussed, with a specific focus on the risk/benefit ratio for each approach and the importance to be compliant with the therapy. Moreover, during these sessions, the patient is invited to actively participate as the “expert,” describing her own personal experience. At the end of the session, an informative booklet will be provided to patients and their family members, summarizing the main aspects discussed during the session.
3. Early warning signs. The third session is focused on the early warning signs of perinatal depression (e.g., changes in the number of sleeping hours; presence of irritability; anxiety; etc.) and on the importance to early detect such signs. In particular, patients are requested to identify, with the support of the mental health professional, their own warning signs and to report them on the “Schedule on early warning signs.” Strategies for preventing or managing crises are discussed and reported in the same schedule.
4. Management of suicidal behaviors. This module is focused on suicidal risk and on the identification of warning suicidal signs. During this module, an *ad-hoc* schedule is provided to participants and family members in order to define a plan to be used in case of necessity.
5. Communication skills sessions are focused on teaching strategies on how to express pleasant or unpleasant feelings, and on how to improve active listening. This module includes the use of role-plays and case vignettes, based on the personal experience of the patients and their family members.
6. Problem solving skills. The last session is focused on teaching problem-solving techniques. Participants are invited to define a problem in their daily routine and to list all potential strategies to solve it. During the session, participants are guided by mental health professional to develop a plan for solving the problem and discuss possible advantages and disadvantages of each possible solution.

Each session lasts about 90 min. Moreover, two or more booster sessions are planned, if needed. Sessions are developed in order to stimulate discussion and interaction among participants. Site and frequency of sessions can be adapted to families' needs and mental health professionals' duties and workloads. The intervention will be carried out at the local mental health center and provided by trained mental health professionals, the time to complete all modules will be between 42 and 60 days. An *ad-hoc* manual has been developed by the research group in order to ensure treatment fidelity. Leaflets and other written materials will be given to patients and family members, when relevant (Table 1).

**Table 1:** Characteristics of the interventions.

**PSYCHOEDUCATIONAL FAMILY INTERVENTION**
Uni-familiar psychoeducational intervention, scheduled every 7–10 days. It consists of six modules:
• Individual and family assessment
• Information on the clinical and epidemiological characteristics of the disorder
• Early warning signs
• Management of suicidal behaviors
• Communication skills
• Problem solving skills
Each session lasts about 90 min. Moreover, two or more booster sessions will be planned, if needed. Sessions are developed in order to stimulate discussion and interaction among participants. Leaflets and other written materials will be given to patients and family members when relevant.
**BEST TREATMENT OPTION (BTO)**
Provided according to the NICE guidelines
• Persistent subthreshold depressive symptoms/ mild to moderate depression: self-help and psychological counseling
• History of severe depression who initially presents with mild depression: pharmacological treatment
• Moderate or severe depression: high intensity psychological intervention, or pharmacological treatments, or an integration of both interventions.

### Control Intervention

Patients allocated in the control intervention will receive the best treatment option (BTO) according to the NICE guidelines (62). At each assessment point, the BTO will be documented through the use of the Pattern of Care Schedule (PCS), an *ad-hoc* schedule filled in by the researcher together with the treating psychiatrist. PCS aims to collect all the information related to the treatments received by the patient. Moreover, patients will continue to be in contact with the treating psychiatrist and whether necessary will receive psychological counseling, both individual, or familiar.

## Training of Mental Health Professionals

Three mental health professionals (at least one will be a psychiatrist) will receive an *ad-hoc* training course for the provision of the experimental intervention. Supervision meetings will be organized during the study period in order to ensure fidelity to the procedure.

## Ethical Issues

This study is being conducted in accordance with globally accepted standards of good clinical practice, in agreement with the Declaration of Helsinki and with national and local regulations. The study investigators ensure that all mental health professionals involved in the study are qualified and informed about the protocol, interventions, and trial-related duties. The study protocol has been submitted to the Ethical Review Board of the University of Campania “Luigi Vanvitelli.”

## Assessment Time and Instruments

Researchers participating to the study are blinded to patient allocation. All patients are assessed at the following time points: baseline (T0); 3 months post-randomization (T1); 6 months post-randomization (T2); 9 months post-randomization (T3); 12 months post-randomization (T4) (Table 2).

**Table 2:** Assessment tools adopted in the study's protocol.

	**Screening phase**	**T0 (baseline)**	**T1 (month 3)**	**T2 (month 6)**	**T3 (month 9)**	**T4 (month 12)**
**ASSESSMENT INSTRUMENT**						
Edinburgh Postnatal Depression Scale (EPDS)	x					
Hamilton Depression Rating Scale (HAM-D)		x	x	x	x	x
Hamilton Anxiety Rating Scale (HAM-A)		x	x	x	x	x
Global Assessment of Functioning (GAF)		x	x	x	x	x
Clinical Global Impression (CGI)		x	x	x	x	x
Manchester Short Assessment of Quality of Life (MANSA)		x	x	x	x	x
Family Assessment Device (FAD)		x	x	x	x	x
Family Coping Questionnaire (FCQ)		x	x	x	x	x
Socio-demographic schedule		x	x	x	x	x
Pattern of Care Schedule (PCS)		x	x	x	x	x

The following questionnaires and schedules will be used during the study:

1. The Edinburgh Postnatal Depression Scale (EPDS) (64) is a simple and short 10-items self-report screening questionnaire initially developed for use in postnatal women to improve detection of postnatal depression. The EPDS allows health professionals to detect women who might need help and require a referral for a full diagnostic assessment. EPDS has satisfactory sensitivity and specificity values and it is also sensitive to changes in severity of depression over time. For the purpose of the present study, the cut-off threshold has been set at ≥ 10 (66). In validation studies, different cut-off points have been found due to clinical, social, cultural and economic diversities (70). The cut-off score for possible depression (i.e., a positive screening result) is ≥13 points but, as suggested by Hewitt et al. (71), a cut-off score ≥ 10 can be used in order to identify patients with both major or minor depression. Moreover, given the observed variability in the performance of the test, the screening procedure could produce both false positives and negatives (72). Therefore, it is essential that this screening tool is complemented by the clinical judgment (73).
2. The Hamilton Depression Rating Scale (HAM-D) (74) is a 17-items questionnaire used to rate the severity of depressive symptoms such as low mood, insomnia, agitation, anxiety and weight loss. The interview and scoring take about 15 min. The score for each item ranges from 0 (not present) to 4 (extreme severity).
3. The Hamilton Anxiety Rating Scale (HAM-A) (75) is a 14-items questionnaire developed to measure the severity of anxiety symptoms, both psychic anxiety (mental agitation and psychological distress) and somatic anxiety (physical complaints related to anxiety). The score for each item ranges from 0 (not present) to 4 (extreme severity).
4. The Global Assessment of Functioning (GAF) (76) is a 100-point rating scale assessing social, occupational, and psychological functioning of adults, with higher scores indicating better level of functioning.
5. The Clinical Global Impression (CGI) (77) scale measures illness severity (CGI-S), global change (CGI-C) and therapeutic response. The CGI-S is rated on a 7-point scale, from 1 (normal) to 7 (the most severely ill patients). The CGI-C scores range from 1 (very much improved) to 7 (very much worse). Treatment response ratings should take into account both the therapeutic efficacy and the treatment-related adverse events, and range from 0 (marked improvement and no side-effects) to 4 (unchanged or worse, and side-effects outweigh the therapeutic effects). Each component of the CGI is rated separately; the instrument does not provide a global score.
6. The Manchester Short Assessment of Quality of Life (MANSA) (78) is a 17-item questionnaire assessing quality of life focusing on satisfaction in twelve aspects of life. For twelve items, the satisfaction is rated on 7-point rating scales, ranging from 1 (“could not be worse”) to 7 (“could not be better”). For five items, the responses are binary (“yes” or “no”). The total MANSA score is the mean of the Likert-item scores.
7. The Family Assessment Device (FAD) (79), based on the McMaster Model of Family Functioning, consists of 7 subscales on family involvement in patient's care (affective involvement, affective responsiveness, behavioral control, communication, problem solving, and roles and general family functioning). Scores range from 1 (“healthy functioning”) to 4 (“unhealthy functioning”).
8. The Family Coping Questionnaire (FCQ) (80) is a self-administered 34-items questionnaire, which has shown a good reliability and external validity. Each item is rated on a 4-level scale, from 1 (“never”) to 4 (“always”). The items are grouped into the following 11 subscales: information on patient's illness; positive communication toward the patient; relatives' maintenance of social interests; patient's involvement in social activities; talking with friends about the patient's condition; coercion; avoidance; resignation; use of alcohol and drugs; collusion; search for spiritual help. The FCQ is widely used for the assessment of coping strategies among relatives of people with severe mental disorders (81, 82).
9. The Pattern of Care Schedule (PCS) (80), a 40-item questionnaire on pharmacological and non-pharmacological treatments as well as on health care access made by the patient. It is compiled by the researcher in collaboration with the patient. If information is inadequate, or if the researcher is not sure about patients' reliability, other sources (e.g., treating physician, relatives, etc.) can be consulted. During the study period, the treating clinician will continue to provide the usual treatment to patient, and–if necessary–to change or adjust the pharmacological regimen. This schedule is used in order to record pharmacological treatments and doses, psychotherapeutic and psychosocial interventions (type of intervention, number of sessions) provided to patients in order to document the BTO.

## Baseline Assessments

At baseline the following information will be collected: (1) sociodemographic data (age, nationality, educational level, marital status, employment status, number of family members, duration of the illness, time in charge at the mental health center (months), number of (voluntary and involuntary) hospitalizations, suicide attempts (numbers); (2) clinical features related to pregnancy (gestational period, previous pregnancies, *in vitro* fertilization, clinical conditions of the fetus, and the pregnant); (3) social context, such as relationship with the partner, family conflicts, socio-economic stressors, history of any psychiatric disorder (Table 3).

**Table 3:** Baseline assessments.

**Sociodemographic data**	**Clinical features related to pregnancy**	**Social context**
• Age	• Gestational period	• Relationship with the partner
• Nationality	• Previous pregnancies	• Family conflicts
• Educational level	• *In vitro* fertilization	• Socio-economic stressors
• Marital status	• Clinical conditions of the fetus and the pregnant	• History of any mental disorder
• Employment status		
• Number of family members		
• Duration of the illness		
• Time in charge at the mental health center		
• Number of hospitalizations		
• Suicide attempts		

## Statistical analyses

### Power Analysis

In order to assess the efficacy of the experimental intervention, a power analysis was performed. As reported by Sockol et al. (83), available psychosocial interventions for perinatal depression have been found to have an overall effect size of 0.65. Therefore, the sample size has been defined selecting 0.65 as desired level of effect size, an α error set at 0.05 and power set at 0.95. Therefore, the total sample will consist of 126 patients, allocated with a 1:1 ratio to the experimental or to the control group (Figure 1).

### Data Analysis

Differences in socio-demographic and clinical characteristics, such as severity of depressive and anxiety symptoms, quality of life, trimester of pregnancy, and personal functioning between the two groups will be evaluated using Chi-square or *T*-test for independent samples, as appropriate. Differences in coping strategies and in family accommodation reported by relatives will be evaluated with the same tests.

In order to assess the efficacy of the experimental intervention, a linear multivariable regression model will be implemented, using as main outcome the score at HAM-D at 3 months post-randomization. Moreover, several confounding variables (such as age, marital status, familiarity for mental disorders, peer support, previous pregnancy, etc.) will be entered in the model. Furthermore, predictors of response at the end of the intervention will be identified.

“Response” is generally defined as a 50% decrease in scores on Hamilton Depression Rating Scale (HAMD) (74). In this protocol, we will consider the score at the 3-month HAM-D compared with the baseline score. Therefore, the continuous variable “3-month HAM-D score” will be transformed in a binary variable (1 = response; 0 = absence of response), and it will be entered in a logistic regression multivariate model in order to identify possible predictors of positive response to the experimental intervention.

In order to evaluate differences in relatives' coping strategies according to patients' clinical features and type of relationship with the patient, a two-level model with fixed independent variables and random intercept, using a maximum likelihood estimation, will be performed. The mean score at the subscales of the FCQ will be entered in the model, adding covariates step by step in order to identify the model with indexes of best fit. The final model will be selected according to the−2 loglikelihood value, and the model with the lowest value will be choose. Estimate, standard error, 95% confidence interval, and *P*-values will be calculated.

The level of significance will be set at *p* < 0.05. All analyses will be performed using the Statistical Package for Social Science software (SPSS), version 18.0 (SPSS).

## Stepwise Procedure

From month 1 to 6, the following phases have been carried out: (1) development of the work plan for data collection; (2) training course for mental health professionals on the interventions; (3) training course for researchers on assessment tools. From month 7 to 12, patients' recruitment will be implemented. From month 7 to 18, interventions will be provided to participating patients. From month 7 to 20, patients' follow-up assessments are being made. From month 20 to 24, statistical analyses will be performed as well as findings will be disseminated through scientific papers, posters and conference participation. A specific plan for dissemination will be developed, including institutional newsletters, press and media release, flyers, training course throughout the national context in order to promote the implementation of the intervention on a large scale. Moreover, users and carers will be involved in the process of dissemination organizing thematic conferences for presenting the characteristics of the intervention.

**Figure 2:**
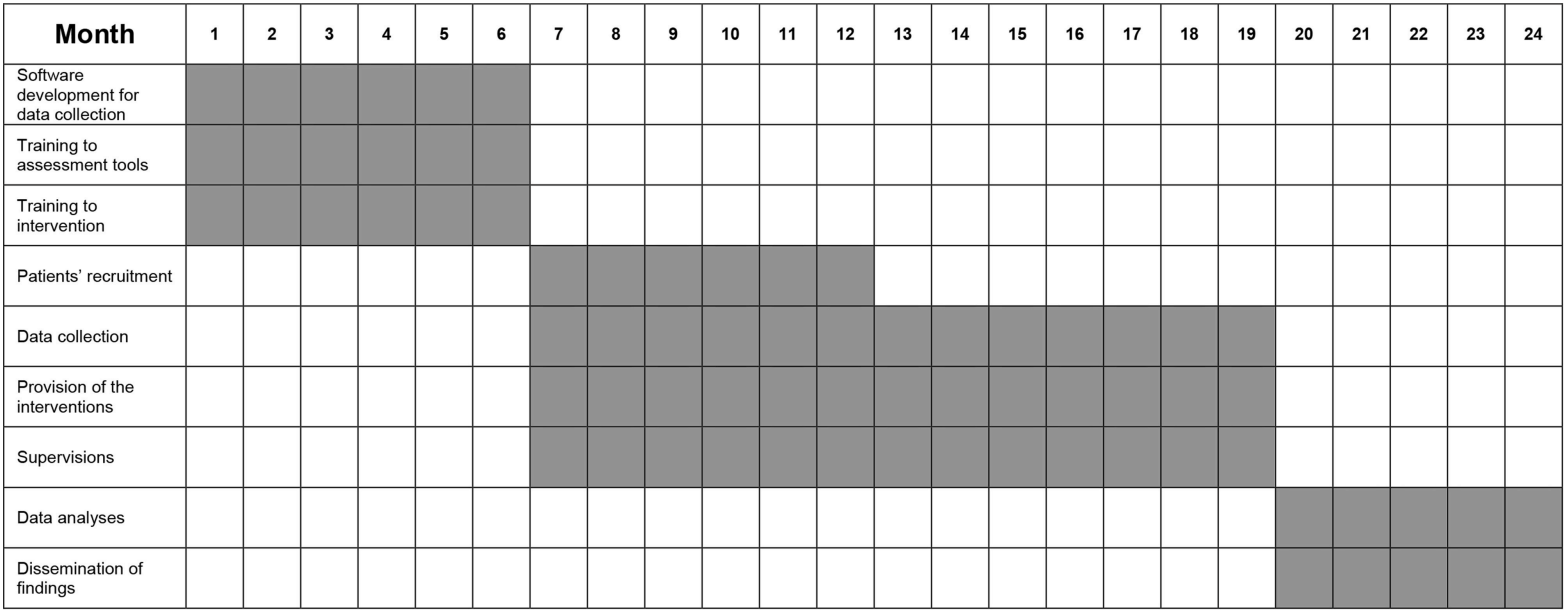
Stepwise procedure.

The stepwise procedure is summarized in Figure 2.

## Anticipated Results

### Primary Outcome

The severity of depressive symptoms, evaluating at the HAM-D, has been selected as primary outcome since it is a reliable index and it has already been used in previous studies on the efficacy of psychosocial interventions (84). In particular, the primary outcome is the reduction of at least 50% of the HAM-D scoring at 3 months post-randomization (T1). The work hypothesis is that BTO plus experimental intervention will be more effective than the BTO alone in reducing the severity of depressive symptomatology.

### Secondary Outcome

The secondary outcomes include an improvement in the severity of anxiety symptoms (evaluating at the HAM-A), of global functioning and personal functioning (evaluated at the CGI and at the GAF, respectively), an improvement in family members' coping strategies (i.e., an improvement in “problem-oriented” strategies reduction); in the level of quality of life (evaluated at the MANSA) and of family functioning.

## Discussion

Pregnancy is a stressful period for women, which can be further complicated by an early postnatal hospital discharge (85, 86). Risk factors for PD include previous mental disorders, low socioeconomic status, being a single mother, general life stress (12), unwanted and unplanned pregnancies (22), conflicts with the partner, lack of support, limited information, and stigma (87).

Despite many scientific associations and task forces, including the United States' Preventive Services Task Force (USPSTF) (88), the National Institute for Health and Care Excellence (62), the Canadian Task Force on Preventive Health Care (CTFPHC) (89) and the American College of Obstetricians and Gynecologists (68), have highlighted the importance of developing screening and treating programs for PD, there are still many unsolved issues, such as efficacy and the availability of those programs, whose efficacy has been demonstrated by RCTs.

One of the main strengths of our study is represented by the adaption of a well-known family psychoeducational model to the treatment of perinatal depression. In particular, the Falloon psychoeducational intervention was developed for the community management of schizophrenia (63, 90) and has been subsequently adapted to the management of major depression (59, 91) and bipolar disorder (57, 92–94). Perinatal depression may represent an ideal target for this kind of interventions, since it is highly dependent from stressful life events and from family context. Moreover, peripartum is a period of women's life in which pharmacological treatments should be used very cautiously and only in a limited situations. Another strength of our study is the rigorous methodology adopted, which will allow us to understand the impact of the experimental intervention on patient's outcome through a randomized controlled approach. Moreover, the main outcome is the reduction of depressive symptoms at 3-months after randomization. This is an ambitious outcome, since many confounding variables can impact on it. In particular, in some cases the childbirth represents itself an event associated with a reduction of depressive symptoms (95), thus biasing the effectiveness of the experimental intervention. Moreover, other contextual factors (such as support by family members, changes in the daily routine following the childbirth, or starting a pharmacological treatment) can have an impact on the long-term outcome. We aim to control for such variables using a statistical procedure, in order to accommodate for the impact of all these confounding variables.

Furthermore, the postnatal psychoeducation programme is suitable for clinical use as it is relatively brief and can be delivered by postnatal unit nurses and midwives after a short period of training.

Another strength of this study is the use of the EPDS as screening measure, which will allow comparisons with data from other international studies.

In order to detect patients affected by both major and minor depression, the cut-off at EPDS of ≥10 has been selected (71) and it will have to be complemented by the clinical judgment (96).

The assessment tools have been selected on the basis of their wide use in clinical practice and in clinical trials (84). Therefore, the adoption of these instruments will give us the opportunity to compare our findings with those of other previous studies. Moreover, the HAM-A and HAM-D are well-known, validated, reliable, and easy to use scales, and have been selected in order to not overburden the researchers and mental health professionals involved in the study with a specific training on the use of assessment tools (84).

Our study has some limitations. The first is the fact that the study will be carried out in one center only, with a consequent reduced generalizability of findings. However, this can be considered as a pilot study representing the basis for a larger multicentric study that our research group is planning.

Another possible limitation is the exclusion from the study of women with psychotic disorders or women with depressive disorders before pregnancy. This methodological choice was due to the need to assess and treat only patients with an onset of depression during pregnancy. Another possible limitation is the lack of a standardized pharmacological treatment for women that will be recruited for the RCT phase. Despite a standardized pharmacological treatment could be useful to assess more precisely the efficacy of the experimental psychoeducational intervention, we decided not to standardize the pharmacological treatment in order to treat women with PD under ordinary conditions. In any case, the pharmacological management of pregnant women will follow the NICE guidelines (62).

## Conclusions

Perinatal depression represents a serious threat for mental health, also considering the detrimental consequences for children. Therefore, it is an ethical imperative to identify new strategies for adequately treat such conditions and reduce the long-term negative impact on the mothers as well as their babies and family members. We hope that the study we will carry on will help to improve the clinical, psychosocial, and family management of perinatal depression.

## Author Contributions

LS, VC, ML, MT, GS, and AF designed the study and wrote the protocol. AF, VDV, ML, and LS organized the training and supervision for mental health professionals. ADC, GF, VC, FZ, and VG performed the literature search. GS developed the plan of statistical analyses. LS, AF, MT, and ML coordinated the study.

### Conflict of Interest Statement

The authors declare that the research was conducted in the absence of any commercial or financial relationships that could be construed as a potential conflict of interest.
